# Effect of *Lycium barbarum polysaccharide* supplementation in non-alcoholic fatty liver disease patients: study protocol for a randomized controlled trial

**DOI:** 10.1186/s13063-021-05529-6

**Published:** 2021-08-26

**Authors:** Lu-Lu Gao, Yu-Xiang Li, Jia-Min Ma, Yi-Qiong Guo, Lin Li, Qing-Han Gao, Yan-Na Fan, Meng-Wei Zhang, Xiu-Juan Tao, Jian-Qiang Yu, Jian-Jun Yang

**Affiliations:** 1 School of Public Health, Xinxiang Medical University, Xinxiang, 453003 Henan China; 2 School of Public Health and Management, Ningxia Medical University, 1160 Shengli Street, Yinchuan, 750004 China; 3 School of Nursing, Ningxia Medical University, 1160 Shengli Street, Yinchuan, 750004 China; 4 Physical Examination Center, People’s Hospital of Ningxia Hui Autonomous Region, 301 Zhengyuan North Street, Yinchuan, 750004 China; 5 Department of Pharmacology, Pharmaceutical Institute of Ningxia Medical University, 1160 Shengli Street, Yinchuan, 750004 China

**Keywords:** Non-alcohol fatty liver disease, *Lycium barbarum polysaccharide*, Prebiotics, Gut microbiota

## Abstract

**Background:**

Non-alcohol fatty liver disease (NAFLD) is the most common chronic liver disease in the world, with a high incidence and no effective treatment. At present, the targeted therapy of intestinal microbes for NAFLD is highly valued. *Lycium barbarum polysaccharide* (LBP), as the main active ingredient of Lycium barbarum, is considered to be a new type of prebiotic substance, which can improve NAFLD by regulating the gut microbiota. The purpose of this study is to evaluate the safety and efficacy of LBP supplementation in modulating gut microbiota for NAFLD patients.

**Methods:**

This randomized, double-blind, placebo-control study will be conducted in the physical examination center of the Ningxia Hui Autonomous Region People’s Hospital. A total of 50 patients with NAFLD confirmed by abdominal ultrasound, laboratory tests, and questionnaire surveys will be recruited and randomly assigned into the control group (maltodextrin placebo capsules) and the intervention group (LBP supplementation capsules) for 3 months. Neither patients, nor investigators, nor data collectors will know the contents in each capsule and the randomization list. The primary outcome measure is the level of ALT concentration relief after the intervention. Secondary outcomes include gut microbiota abundance and diversity, intestinal permeability, patient’s characteristic demographic data and body composition, adverse effects, and compliance from patients.

**Discussion:**

LBPs are potential prebiotics with the property of regulating host gut microbiota. Our previous studies have documented that LBP supplement can improve the liver damage and the gut microflora dysbiosis in NAFLD rats. This treatment would provide a more in-depth understanding of the effect of this LBP supplementation.

**Trial registration:**

Chinese Clinical Trial Register, ChiCTR2000034740. Registered on 17 July 2020.

**Supplementary Information:**

The online version contains supplementary material available at 10.1186/s13063-021-05529-6.

## Background

Non-alcoholic fatty liver disease (NAFLD) is characterized by the presence of fat accumulation more than 5% in hepatocytes, which is not due to hepatitis B virus (HBV) or hepatitis C virus (HCV) infection and excessive alcohol consumption. It is a chronic and wide spectrum of liver disease, involving a complex progression from simple steatosis to non-alcoholic steatohepatitis (NASH) and then to cirrhosis, hepatocellular carcinoma, even lead to liver transplantation and death [1]. Over the past few decades, the estimated incidence and prevalence of NAFLD has risen dramatically globally due to dramatic changes in lifestyle and the improvement of living standards. According to a systematic review and meta-analysis, no matter what diagnostic method was used, the global prevalence of NAFLD was 29.62% [2, 3]. NAFLD primarily associated with low physical activity levels, central obesity, diabetes, hypertension, and metabolic syndrome has become an important public health issue [4]. However, the mechanism of pathogenesis and progression of NAFLD is multifactorial and complex and there are no approved pharmacological therapies for the treatment of NAFLD up to now [5].

It is well known that the liver and intestine have anatomical links through the hepatic portal system, the gut microbiota and its metabolites may be involved in the pathogenesis of liver disease, and the gut-liver axis is increasingly recognized as a key player [6]. The gut epithelium is a natural physical barrier that prevents the translocation of detrimental gut bacteria and their metabolic by-products into the blood [7]. It was documented that high fat diets could lead to microbiota dysbiosis and the gut barrier dysfunction, which increase intestinal permeability and allow the bioproducts of gut microbiota such as LPS to enter the portal circulation [8]. Lipopolysaccharide (LPS), the active component of endotoxin, is the main gut-derived metabolites that may be involved in the pathogenesis of NAFLD. Serum increased LPS combined the Toll-like receptor 4 (TLR-4) complexes and subsequently stimulating the production of inflammatory reaction and insulin resistance in the liver [9]. Therefore, gut microbiota is an important environmental factor involving the pathogenesis of NAFLD.

Probiotics are defined by the Food and Agriculture Organization and World Health Organization (FAO/WHO) as a collection of living microorganisms that are beneficial to the host metabolism [10, 11]. Several studies have reported the probiotic supplementation could restore the gut microbiota structure and intestinal barrier integrity [12]. Ma et al. [13] performed a meta-analysis evaluating the effect of probiotics on NAFLD, demonstrating that probiotics significantly improved NAFLD by reducing the levels of serum ALT, AST, LPS, and liver TNF-α, TLR4. Studies from Li et al. [14] suggested that probiotics could enhance the barrier integrity of epithelial cells and restore normal gut flora structure and proportion, which reduces the liver injury of NAFLD. *Lactobacillus* and *Bifidobacterium* are the most commonly used symbiotic or probiotics, which can inhibit harmful bacteria growth and have a wide range of benefits on human health [15]. They are naturally contained in common foods, fruits, and tea. In conclusion, regulating enteric flora by probiotics or prebiotics may be a new therapeutic approach for the prevention and treatment of NAFLD [16].

Lycium barbarum (Solanaceae), also called Goji berry or wolfberry, is a well-known traditional Chinese herbal medicine and functional food for many years. It was found mainly in northwest China and has gained an increasing popularity due to its effect in nutritional and health-promoting properties. Goji berries are rich in polysaccharides and one of the most functional active components of wolfberry is *Lycium barbarum polysaccharides* (LBPs) [17]. In recent years, the health benefits of LBP have been reported more and more frequently, such as antioxidant, lipid-lowering, hepatoprotective, neurological protective, and vision-protective. Furthermore, LBP exerts promote effects on the proliferation of “good” bacterium that can be used as a new kind of probiotics which was detected by Fang Zhou et al. [18]. Recent study from Zhu et al. [19] also found that LBPs from Goji berry have prebiotic activities which can stimulate beneficial bacteria growth in vitro, also balancing the microbial composition in gut, enhancing the bacteria concentration and immunity in mice. These results indicate that LBPs are potential prebiotics with the property of regulating host gut microbiota. Taking these advantages as listed above into account, the LBP protective role on many diseases for example type 2 diabetes mellitus (T2DM), liver injury, Parkinson’s disease, and Alzheimer’s disease has been confirmed [20]. However, little is known about whether LBP is a probiotic on the pathogenesis and progression of NAFLD. Considering the positive results of the studies of probiotic on liver injury and there is no specific and effective drug for NAFLD treatment, the hepatoprotective effect of LBP involving NAFLD deserves further study.

In this study, we postulate that LBP can become an adjunct therapeutic option for NAFLD through modulating the gut microbiota. In this context, we conducted a randomized controlled clinical trial to evaluate whether LBP supplementation can improve gut flora disturbance and liver injury of NAFLD.

## Methods/design

### Aim

The primary aim of this trial is to determine the efficacy of LBP supplements in reducing hepatic injury in patients with NAFLD compared to the placebo control group. Secondary objectives of this study include investigating the effects of supplementation with LBP on the gut microbiota, intestinal barrier function, body composition, inflammatory cytokines, and the acceptance and safety for the intervention.

### Study design

**Fig. 1:**
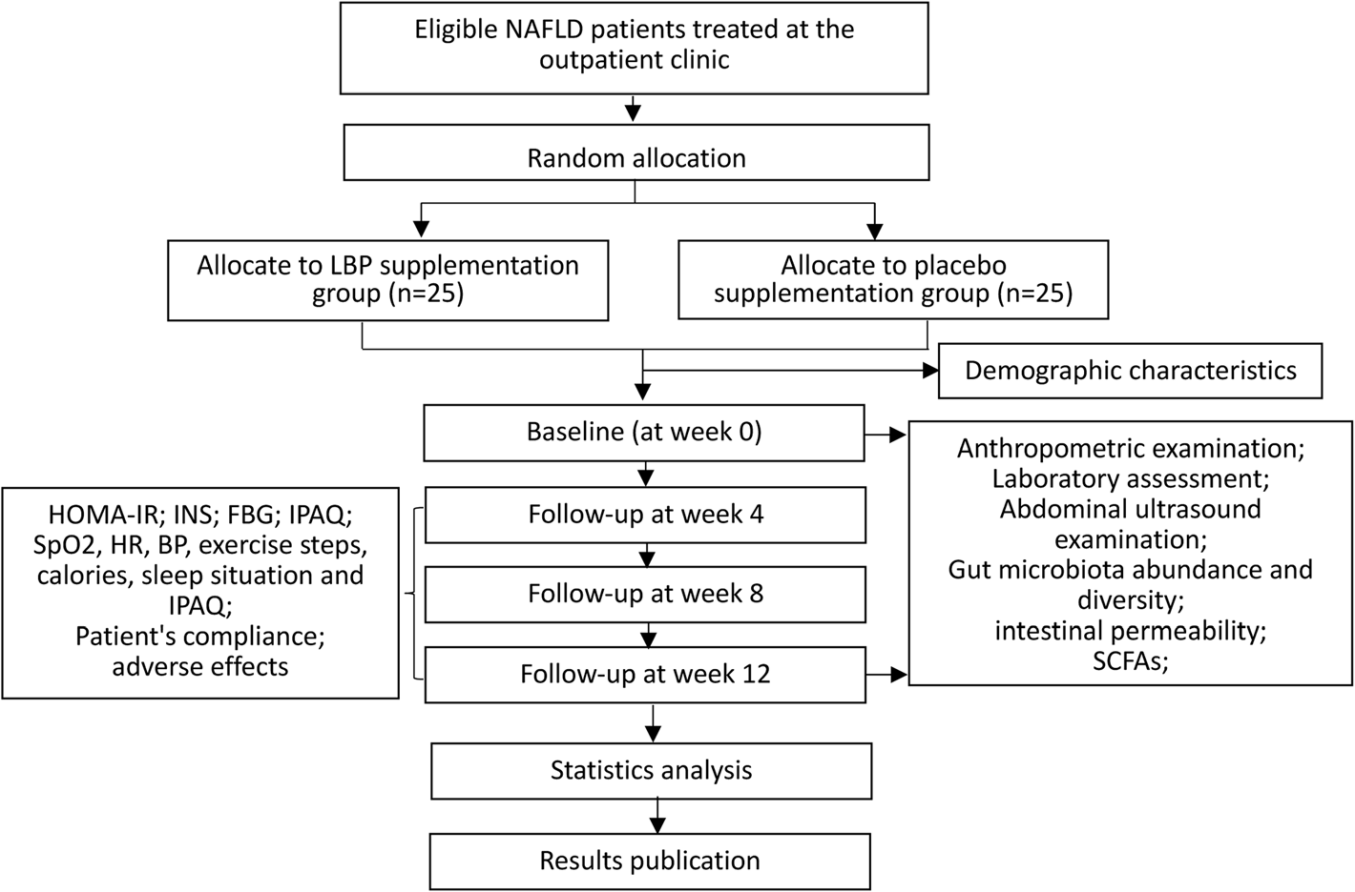
Study design. NAFLD: non-alcoholic fatty liver disease; HOMA-IR, homeostasis model assessment for insulin resistance; INS, insulin; FBG, fasting blood glucose; IPAQ, International Physical Activity Questionnaire-Short Form; SpO_2_, oxygen saturation; BP, blood pressure; HR, heart rate; SCFAs, short chain fatty acids

**Fig. 2:**
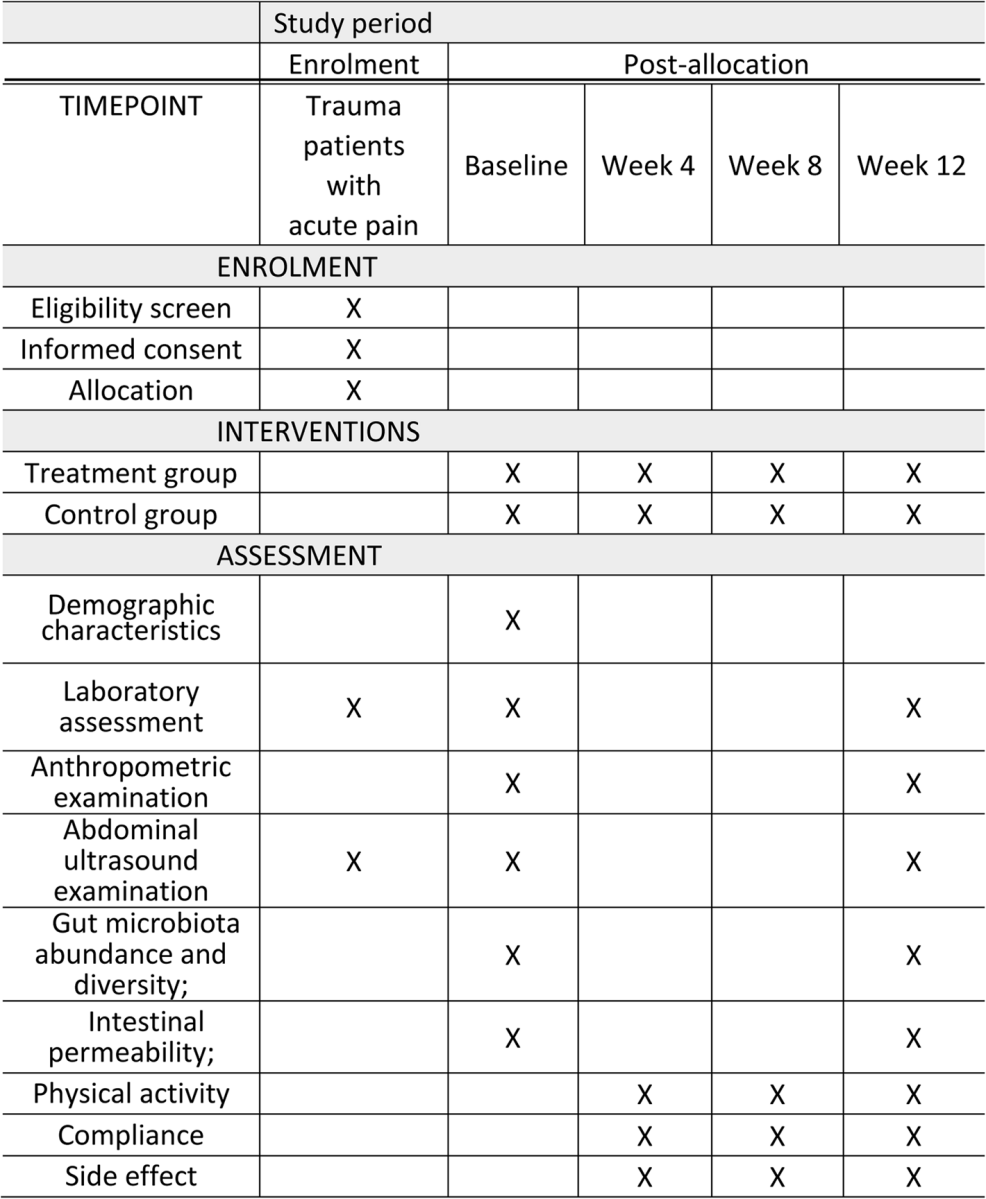
SPIRIT figure: schedule of enrolment, interventions, and assessments

This is a single-center, two-group (1:1), prospective, randomized, double-blind, placebo-control study. The present protocol was designed in accordance with the Standard Protocol Items: Recommendations for Interventional Trials (SPIRIT) guidelines and completed the SPIRIT Checklist [21] (Additional file 1). Additionally, the whole study design is illustrated in Fig. 1 and a SPIRIT figure is presented in Fig. 2.

This randomized controlled clinical trial has been approved (2019-329) by the Institutional Ethics Committee of Ningxia Medical University, and it has been registered at the Chinese Clinical Trial Registry (ChiCTR2000034740).

### Study participants

All patients with NAFLD treated at the physical examination center of the Ningxia Hui Autonomous Region People’s Hospital will be recruited and invited to participate. The specific inclusion criteria are (1) subjects diagnosed with NAFLD through the abnormal liver enzymes and ultrasonography, (2) aged 45~59 years, and (3) willing to participate in the study and sign informed consent. Patients will be excluded if they have (1) acute or chronic viral hepatitis, drug-induced liver disease, liver cirrhosis, or other causes of chronic liver disease; (2) alcohol consumption > 140 g/week in men or > 70 g/week in women over the past 6 months; (3) intestinal diseases or other chronic inflammation; and (4) diabetes or uncontrolled cardiovascular disease. Other exclusion criteria include regular intake of probiotic supplements or food within 3 months, pregnancy, and use of medications such as corticosteroid drugs, immunosuppressants, and antibiotics.

### Intervention

According to the inclusion and exclusion criteria, all eligible patients will be introduced to the study. After investigators comprehensively review the inclusion and exclusion criteria and explain the purpose, benefits, and potential risks of the study, patients who agree to participate will be asked to sign an informed consent form (Additional file 2) and then be included in the study. At the baseline, participants will be randomized to allocate an intervention group or a control group. Patients assigned to the intervention group will receive an LBP supplementation capsule and those assigned to the control group will receive an identical appearance capsule containing maltodextrin as the placebo. In this study, the capsules are provided by Ningxia Wofu Bairui Biological Food Engineering Co., Ltd. The concentration of LBP supplementation capsules is 40% and the specification is 0.75 g per capsule. According to the recommended amount, patients will be instructed to take two capsules daily for 12 weeks and not to change their diet or daily physical activity. Follow-up assessments will be performed every 4 weeks at weeks 4, 8, and 12 after randomized allocation. The trained researcher will successively include patients until the target sample size is reached and the project manager will assign participants to interventions according to the randomization list. In general, both LBP supplementation and maltodextrin placebo are considered safe interventions without severe side effects for participants [20]. If any undesirable event occurs (bleeding, allergic and anaphylactic reactions, interactions with other prescriptions, etc.) or the participant requests to withdraw, the investigator can discern whether the trial should be continued. All reasons for terminating the trial will be recorded by the data collector.

There are many strategies that will be used to assess and improve patient compliance. Before administering the intervention, the baseline data of the participants will be generated and gathered. The investigator will detail the duration of the intervention, possible adverse effects, and provide capsules for a period of 4 weeks. The adherence will be assessed by checking the number of remaining capsules and a capsule intake questionnaire at each follow-up visit. At the same time, patients will obtain another set of capsules (every 4 weeks).

### Randomization, allocation concealment, and blinding

Patients meeting the inclusion criteria will be randomly assigned to the intervention group and control group at a 1:1 allocation ratio after recruitment. The randomly assigned list will be computer-generated (using the website: http://randomization.com) and executed by a statistician who is not clinically involved in this study. The randomization list will be kept in a sealed, opaque envelope by the project manager who is in charge of the capsule distribution, and inaccessible to anyone else. Therefore, except for the project manager, the investigators, the data collectors, and the patients are blind to the patient’s allocation treatment and the composition of each capsule supplement. Other researchers will know the patient’s allocation and each capsule supplement composition only after the completion of this study. If needed, the investigators will be informed which supplements each patient received. After the trial is completed, participants in the control group will have the opportunity to participate in a 30-day LBP supplement program.

### Measurement

The data collector will record the patients’ demographic data, diagnostic methods, and clinical diagnosis and the participant’s anthropometric examination, laboratory assessment, and imaging. Other information regarding insulin resistance, gut microbiota and intestinal permeability, physical-activity, patient compliance, and adverse events will be noted by the researcher.

### Clinical evaluation

Clinical evaluation including patient’s characteristic demographic data (sex, age, nationality, address, telephone number) and anthropometric examination such as height, weight, fat-free weight, BMI, body fat, body fat ratio, waist-hip ratio, and other indicators. The anthropometric examination will be measured with a body composition analyzer (InBody, InBody770). InBody770 is a body composition analyzer of direct segmental multi-frequency bioelectrical impedance analysis (DSM-BIA) method, which is fast, non-invasive, and accurate. The patient demographic data will be carefully assessed and recorded by the investigator before the intervention. The anthropometric results will be evaluated at the beginning of the intervention and at the last follow-up visit.

### Laboratory assessment and imaging

Laboratory investigations include liver enzymes (γ-glutamyltransferase (GGT), alanine aminotransferase (ALT), and aspartate aminotransferase (AST)); biochemical markers of lipogenesis such as triglycerides (TG), total cholesterol (TC), LDL-cholesterol, HDL-cholesterol, and free fatty acids (FFA); and inflammatory factors (IL-6, IL-1β, TNF-α, MCP-1). At baseline and 12 weeks, the blood sample will be collected for laboratory evaluation after a 12-h overnight fasting. Liver enzymes and lipid profiles (TG, TC, LDL-cholesterol, HDL-cholesterol) will be measured by an automatic biochemistry analyzer. The levels of FFA will be assayed using an FFA assay kit (Nanjing Jiancheng Bioengineering Institute, Nanjing, China). Gas chromatography-mass spectrometer (7890B-5977A GC/MSD, Agilent, USA) will be used for the short chain fatty acids (acetic acid, propanoic acid, butyric acid, isobutyric acid, N-valeric acid, isobutyric acid, caproic acid) analysis. The concentration of inflammatory factors will be determined by enzyme-linked immunosorbent assays (ELISA) kit (Elabscience Biotechnology Institute, China). All laboratory assessments will be performed in the same laboratory by using standard laboratory methods. At the beginning and the end of the intervention period, ultrasound examination of the abdomen will be performed on all patients, and the hepatic steatosis will be noted and graded.

### Insulin resistance

The homeostasis model assessment for insulin resistance (HOMA-IR) index will be used to determine the degree of insulin resistance of individuals. It will be calculated by using the formula: [fasting plasma insulin (mU/L) × fasting plasma glucose (mmol/L)]/22.5. All individuals will be advised to fast overnight before blood sample collection. The blood sample collected in the EDTA anticoagulant tubes will be centrifuged and plasma stored at -80 °C for a series of tests. Fasting plasma insulin will be detected by using an ELISA kit (Elabscience Biotechnology Institute, China). Fasting plasma glucose will be instantly measured by the One Touch Ultra 2 blood glucose meter (LifeScan, USA). The testing of insulin and glucose will be performed on each patient at weeks 0 and 12 follow-up assessments.

### Gut microbiota and intestinal permeability

The stool (take two samples every time) will be collected at the baseline before the intervention and the end of 12 weeks intervention for the gut microbiota analysis. The participants will be instructed to collect, store, and transport the fecal samples on proper methods. A convenient specimen collection box will be provided to all patients before the beginning of the intervention. They will be advised to take 2–3 g of fecal samples each time and then immediately store the samples into their own freezer (at least -20 °C). Afterwards, the individuals will notify the researchers to transfer the samples to a -80 °C refrigerator until analysis. For the gut microbiota assessment, total DNA will be extracted from 150 mg of fecal with the QIAamp DNA mini kit (Qiagen, Valencia, CA, USA). The purity and concentration of DNA will be detected by gel electrophoresis for sequencing and bioinformatics analysis. Raw reads will be filtered to remove adaptors and low-quality and ambiguous bases, and then paired-end reads will be added to tags by Fast Length Adjustment of Short reads program (FLASH, vl.2.11) to get the tags. The tags will be clustered into OTUs with a cutoff value of 97% using UPARSE software (v7.0.1090) and chimera sequences will be compared with the Gold database using UCHIME (v4.2.40) to detect. The sequencing data will be analyzed by QIIAME v1.8.0. In addition, the short chain fatty acid (SCFA) concentrations will be determined in the fecal supernatant using the gas chromatograph-mass spectrometer (Agilent 7890B-5977A, USA) for sample analyzing and using ChemStation software (Agilent, USA) for data handling.

The intestinal barrier function and permeability will be determined by the concentration of serum claudin-3 and LPS in plasma. As described above, the blood samples will be used to evaluate the changes of claudin-3 and LPS concentration before and after the intervention. According to the manufacturer’s instructions, serum biomarkers claudin-3 will be measured by ELISA, and the LPS will be performed through the limulus test method.

### Physical-activity

Physical activity will be assessed by using the International Physical Activity Questionnaire-Short Form (IPAQ) [22] at 0, 4, 8, and 12 weeks. They are instructed not to change the current level of physical activity throughout the study. The participants will be provided with a smart activity tracker (Xiaomi, China), which could monitor blood oxygen saturation, heart rate, blood pressure, exercise steps, calories, and sleep situation. Participants will be instructed to wear monitors at least 5 days a week and the data on the monitor worn by the participants will be recorded at each follow-up. It will be used to assess how much time participants spend on physical exercise each week.

## Outcome measures

### Primary outcome measure

The primary outcome measure will be a significant ALT concentration relief after the intervention, which is assessed and recorded by the researchers according to the results of laboratory assessment.

### Secondary outcome measures

The following is the secondary outcome:

1. Gut microbiota abundance and diversity assessed by metagenomic sequencing method.
2. Intestinal permeability determined by the concentration of claudin-3 and LPS.
3. Patient’s characteristic demographic data by investigating and the results of laboratory assessment and the body composition evaluated by anthropometry.
4. Patient’s compliance and adverse effects.

### Sample size calculation

Based on the previous study results of supplementing synbiotics in NAFLD, this study took the significant reduction of ALT concentration as the main outcome indicator for sample size calculation. According to the research of Eslamparas et al. [23], it was found that the concentration of ALT decreased 11.3 and 4.4 in the intervention and control groups respectively. It aimed to determine an appropriate target sample size that would provide a 90% power (*β* = 0.10) for two-tailed testing with 5% type-1 error rate of the measure. Using the PASS 22.0 software, we calculated a minimum requirement of 42 patients for total. In practice, we decided to enroll a total of 50 participants (*n* = 25 per group), taking into account the 20% drop-out rate before the end of the study.

### Recruitment

This study will be conducted in the physical examination center of the Ningxia Hui Autonomous Region People’s Hospital. The number of NAFLD patients admitted to the physical examination center each year is enough to meet the sample size of this study. We will recruit participants through the WeChat public platform, posters, telephone visit, and the medical staff introductions.

For patients who wish to participate in the study, researchers will elaborate on the purpose, methods, possible risks, and rights of participants. If the patient is fully aware of the study and agrees to participate and meets the inclusion criteria, the investigator will ask the patient to sign an informed consent form.

### Participant retention and withdrawal

Once the participants are registered, the research team has the responsibility to achieve a low rate of loss to intervention. Before the start of the intervention, investigators will conduct education, detailing the duration of the intervention and possible adverse effects. During the intervention and follow-up period, the trained researchers will remind the subjects to take the capsules, fill in the capsule taking record form, ask if there is any uncomfortable, and give psychological support to participants.

All patients will be informed that they have the right to withdraw from the study at any time during the intervention and the Data Monitoring Committee (DMC) will discuss and analyze the reasons for dropouts with data collectors, which will be documented in a standardized proforma.

### Data collection, management, and confidentiality

Paper material of the original test data will be stored in a locked filing cabinet in the office of the Ningxia Medical University. All data will be fed into the computer by two researchers, and access passwords will be set to keep the data secure. Personal identifiable subject information related to the data will be replaced with anonymous numbers. In order to ensure the quality of data, an independent clinical research assistant will review the original data every month to check whether the data is correct and complete. The authors will follow the guidelines recommended by the International Committee of Medical Journal Editors (ICMJE).

### Data monitoring

A DMC, which is independent from the sponsor and any competing interests, will be set up at the same time the research project is identified refer to the DMC charter [21, 24]. The DMC includes a chief nutritionist, an endocrinologist, a chief nurse, and a statistical specialist who will serve as the DMC’s chair. During the implementation of the study, members of the DMC will review the original test data regularly, check the standardization of the study, ensure the completeness and accuracy of the data, and guarantee the credibility and dependability of the study results. If the study is found to be out of order, the DMC may make corrective recommendations or stop the test.

Any modifications of this protocol and changes to eligibility criteria or outcomes will be communicated with the DMC, project implementer, trial participants, and research ethics committee review board. In regular DMC meetings, they discuss project issues, modify the study design, and convey the protocol changes to the project implementer.

### Auditing

The trial funder will audit and review the source data to verify the accuracy, completeness, and safety of the data annually. What is more, the members of DMC and the responsible researchers will inspect the original documents, the fund utilizing, and the medical records of patients as well.

### Data analysis

All patients randomized should be included in the analysis and missing data will be handled using multiple imputation. The test data will be analyzed by a statistical expert using SPSS version 22.0 (Chicago, IL, USA). The statistical expert is not involved in the experiment and is only responsible for the analysis of the data. We will use descriptive statistical methods to measure demographics and baseline clinical features. The categorical variables will use the chi-square test or Fisher’s exact test for analysis and are reported as numbers and percentages. The numerical variables will be reported as mean±standard deviation or medians with interquartile ranges (IQR) comparing by Student’s *t* test or the Mann-Whitney *U* test. Ranked data will be analyzed using the rank-sum test. Follow-up data will be compared using the repeated measures analysis of variance (ANOVA). A multiple linear regression method was used to establish a linear equation of multiple linear regression to detect the interaction between the amount of physical activity and the efficacy of LBP supplements.

The alpha and beta diversity of the gut microbiota will be estimated by MOTHUR and QIIME at the OUT level, respectively. Sample cluster will be conducted by QIIME based on UPGMA. KEGG and COG functions will be predicted using the PICRUSt software. Barplot and heatmap of different classification levels will be plotted with T package v3.4.1 and R package “gplots”, respectively. Cases with missing values in the resulting parameters will be excluded from the analysis. All results are statistically significant with *P*<0.05.

## Discussion

Although witnessed major efforts in the prevention and treatment of NAFLD over the past decades, it remains a worrying disease and prevalent worldwide with the rise of obesity and diabetes [25]. It has been recognized as the most common cause of chronic liver disease and may lead to cirrhosis and liver carcinoma [26]. Noticeably, NAFLD is predicted to be the leading cause of liver transplantation in the next 20 years, contributing to liver-related morbidity and mortality [4]. As of today, despite the tremendous progress made in studying the pathogenesis of NAFLD, the main factors leading to the evolution of NAFLD from simple steatosis (NAFL) to NASH have been largely unknown. However, there is still a lack of approved or effective pharmacological treatment for patients with NAFLD in clinical practice, and lifestyle modifications remain the main strategies [27]. Take the high incidence and serious health risks of NAFLD into consideration, the lack of effective therapy has attracted great concern. Recently, multiple researches have demonstrated that the gut microbiota is closely related to the development and progression of NAFLD through transmitting its own components or metabolites. According to the study of Bäckhed et al. [28], the normal cecum microbiota of conventionally raised mice was colonized into germ-free (GF) C57BL/6 J mice, and it was found that, despite the food intake reduced, the body fat content significantly increased along with insulin resistance occurred after 14 days. Their further research showed that [29], compared with conventional mice with gut microbiota, the GF mice were protected from obesity after eating a Western-style, high-fat, and high-sugar diet. NAFLD could be transmitted to GF mice through fecal microflora transplantation (FMT), which indicates that gut microbes are directly involved in the development of NAFLD. In clinical studies, Miele et al. [30] found that in contrast to healthy volunteers, the intestinal permeability in NAFLD patients was significantly improved, which was related to the increased prevalence of small intestinal bacterial overgrowth (SIBO). These results were consistent with Harte et al. [31], who confirmed that circulating endotoxin levels in NAFLD and NASH patients were higher than in healthy controls. Taken together, gut microbes are considered as the key factor contributing to the development of NAFLD. In view of the results of animal studies and clinical trials, using prebiotics or synbiotics to regulate intestinal flora could become a novel therapeutic target and strategy for the prevention and treatment of NAFLD [32].

At present, there are sparse researches on the application of probiotics in patients with NAFLD. This randomized, double-blind, placebo-controlled study will describe the characteristic of gut microbiota in the progression of NAFLD and the protective effect of LBP supplementation on NAFLD patients. Considering the limited study in this field, the generalization of prebiotics in NAFLD patients still needs high-quality clinical trials to confirm. We believe that the work in this article will help us understand the mechanisms and the therapeutic effects of LBP supplementation on NAFLD. If this treatment proves to be beneficial, it could provide clinical evidence of a potential low-risk treatment option for NAFLD patients and has a significant impact on the gut microbiota targeted therapy for managing NAFLD.

## Trial status

The study is in the patient recruitment phase. This is version 2.0 of the protocol, dated July 30, 2020. Recruitment started in August 2020 and is predicted to end in November 2020.

## Supplementary Information

**Additional file 1.** SPIRIT 2013 Checklist: Recommended items to address in a clinical trial protocol and related documents.

**Additional file 2.** Informed consent form.

**Additional file 3.** Project Approval Notice of National Natural Science Foundation of China and Related Matters. (Project approval number: 81660537).

**Additional file 4.** Project Approval Notice of National Natural Science Foundation of China. (Project approval number: 82060597).

## Data Availability

After this study is complete, the final trial dataset and statistical codes will be available from the corresponding authors upon reasonable request, except for participants’ personal information. The results will be published in peer-reviewed journals. Findings will be shared with the participants, healthcare workers, the general public, and relevant departments through open-access articles, public talks, conferences, and final reports.

## Abbreviations

NAFLD: Non-alcoholic fatty liver disease
HBV: Hepatitis B virus
HCV: Hepatitis C virus
NASH: Non-alcoholic steatohepatitis
LPS: Lipopolysaccharide
TLR-4: Toll-like receptor 4
FAO/WHO: Food and Agriculture Organization and World Health Organization
LBPs: Lycium barbarum polysaccharides
T2DM: Type 2 diabetes mellitus
SPIRIT: Standard Protocol Items Recommendations for Interventional Trials
GGT: γ-glutamyltransferase
ALT: Alanine aminotransferase
AST: Aspartate aminotransferase
TG: Triglycerides
TC: Total cholesterol
FFA: Free fatty acids
ELISA: Enzyme-linked immunosorbent assays
HOMA-IR: Homeostasis model assessment for insulin resistance
SCFAs: Short chain fatty acids
IPAQ: International Physical Activity Questionnaire-Short Form
ICMJE: International Committee of Medical Journal Editors
DMC: Data Monitoring Committee
ANOVA: Analysis of variance
IQR: Interquartile ranges
NAFL: Simple steatosis
GF: Germ-free
FMT: Fecal microflora transplantation
SIBO: Small intestinal bacterial overgrowth
