# Correction to: Effect of *Lycium barbarum* polysaccharide supplementation in non-alcoholic fatty liver disease patients: study protocol for a randomized controlled trial

**DOI:** 10.1186/s13063-021-05649-z

**Published:** 2021-09-30

**Authors:** Lu-Lu Gao, Yu-Xiang Li, Jia-Min Ma, Yi-Qiong Guo, Lin Li, Qing-Han Gao, Yan-Na Fan, Meng-Wei Zhang, Xiu-Juan Tao, Jian-Qiang Yu, Jian-Jun Yang

**Affiliations:** 1 School of Public Health, Xinxiang Medical University, Xinxiang, 453003 Henan China; 2 School of Public Health and Management, Ningxia Medical University, 1160 Shengli Street, Yinchuan, 750004 China; 3 School of Nursing, Ningxia Medical University, 1160 Shengli Street, Yinchuan, 750004 China; 4 Physical Examination Center, People’s Hospital of Ningxia Hui Autonomous Region, 301 Zhengyuan North Street, Yinchuan, 750004 China; 5 Department of Pharmacology, Pharmaceutical Institute of Ningxia Medical University, 1160 Shengli Street, Yinchuan, 750004 China

**Correction to: Trials 22, 566 (2021)**

**https://doi.org/10.1186/s13063-021-05529-6**

Following the publication of the original article [1], we were notified that the first affiliation of the first author was incorrect.

- Originally published affiliation: School of Nursin, Shandong First Medical University (Shandong Academy of Medical Sciences), Jinan 250000, Shandong, China
- Corrected affiliation: School of Public Health, Xinxiang Medical University, Xinxiang 453003, Henan, China

The original article has been corrected.
