# Supplementary material for: Effect of Lycium barbarum polysaccharide supplementation in non-alcoholic fatty liver disease patients: study protocol for a randomized controlled trial
Source: Trials. 2021 Aug 26;22:566. doi: 10.1186/s13063-021-05529-6 (PMC8439032; doi:10.1186/s13063-021-05529-6)
Supplement: Supplementary file 2 — Additional file 2. Informed consent form. [file 13063_2021_5529_MOESM2_ESM.pdf]

Informed Consent Form

Dear Madam/Sir

We are going to carry out a clinical trial of "Effect of Lycium barbarum polysaccharide supplementation in non-alcoholic fatty liver disease patients: study protocol for a randomized controlled trial". You may be eligible for this trial. Therefore, we invite you to participate in the trial. The sponsor of the trial is Ningxia Medical University, and the main investigators are Yang Jianjun and Gao Lulu.

This informed consent form will introduce you to the purpose, procedures, benefits, risks, inconveniences or discomforts you will bear, and the main items of the trial. It will also introduce you to other treatments that you can choose from And you have the right to withdraw from the research at any time. Please read carefully and make a cautious decision whether to participate in the study. When the researcher explains and discusses the informed consent form to you, you can ask questions and ask to explain the information at any time. You can make a decision after discussing it with your family, friends and your doctor. Your signature will not deprive you of any legal rights and interests. The original signed informed consent form will be kept with the investigator, and the other copy will be kept by you.

1. Background

Non-alcoholic fatty liver disease (NAFLD) is characterized by the presence of fat accumulation more than 5% in hepatocytes, which is not due to hepatitis B virus (HBV) or hepatitis C virus (HCV) infection and excessive alcohol consumption. It is a chronic and wide spectrum of liver disease, involving a complex progression from simple steatosis to non-alcoholic steatohepatitis (NASH) and then to cirrhosis, hepatocellular carcinoma, even lead to liver transplantation and death. Over the past few decades, the estimated incidence and prevalence of NAFLD has risen dramatically globally due to dramatic changes in lifestyle and the improvement of living standards. NAFLD primarily associated with low physical activity levels, central obesity, diabetes, hypertension and metabolic syndrome, has become an important public health issue. However, the mechanism of pathogenesis and progression of NAFLD is multifactorial and complex and there are no approved pharmacological therapies for the treatment of NAFLD up to now.

Lycium barbarum (Solanaceae), also called Goji berry or wolfberry, is a well-known traditional Chinese herbal medicine and functional food for many years. It was found mainly in northwest China and has gained an increasing popularity due to its effect in nutritional and health-promoting properties. Goji berries are rich in polysaccharides and one of most functional active components of wolfberry are *Lycium barbarum Polysaccharides* (LBPs). In recent years, the health benefits of LBP have been reported more and more frequently, such as antioxidant, lipid-lowering, hepatoprotective, neurological protective and vision-protective. Furthermore, LBP exerts a promote effects on the proliferation of “good” bacterium can be used as a new kind of probiotics

was detected by Fang Zhou et al. Recent study from Zhu et al. also found LBPs from Goji berry have prebiotic activities which can stimulate beneficial bacteria growth in vitro, also balancing the microbial composition in gut, enhancing the bacteria concentration and immunity in mice. These results indicate that LBPs are potential prebiotics with the property of regulating host gut microbiota. However, little is known about whether LBP as a probiotic on the pathogenesis and progression of NAFLD. Considering the positive results of the studies of probiotic on liver injury and there is no specific and effect drug for NAFLD treatment, the hepatoprotective effect of LBP involving NAFLD deserves further study.

In this study, we postulate that LBP can become an adjunct therapeutic option for NAFLD through modulating the gut microbiota. In this context, we conducted a randomized controlled clinical trial to evaluate whether LBP supplementation can improve gut flora disturbance and liver injury of NAFLD.

2.Aim

The primary aim of this trial is to determine the efficacy of LBP supplements in reducing hepatic injury in patients with NAFLD compared to the placebo control group. Secondary objectives of this study include investigating the effects of supplementation with LBP on the gut microbiota, intestinal barrier function, body composition, inflammatory cytokines as well as the acceptance and safety for the intervention.

3. Inclusion and exclusion criteria:

This randomized controlled clinical trial has been approved (2019-329) by the Institutional Ethics Committee of Ningxia Medical University and it has been registered at the Chinese Clinical Trial Registry (ChiCTR2000034740).

All patients with NAFLD treated at the physical examination center of the Ningxia Hui Autonomous Region People's Hospital will be recruited and invited to participate. The specific inclusion criteria are: 1) subjects diagnosed with NAFLD through the abnormal liver enzymes and ultrasonography; 2) aged 45~59 years; and 3) willing to participate in the study and sign informed consent. Patients will be excluded if they have 1) acute or chronic viral hepatitis, drug-induced liver disease, liver cirrhosis or other causes of chronic liver disease; 2) alcohol consumption >140 g/week in men or > 70 g/week in women over the past 6 months; 3) intestinal diseases or other chronic inflammation; 4) diabetes or uncontrolled cardiovascular disease. Other exclusion criteria include regular intake probiotic supplements or food within 3 months, pregnancy, use of the medicine such as corticosteroid drugs, immunosuppressant, and antibiotic.

4. If you participate in the research, you will need to do the following work

(1) Before you are selected for the study, you will undergo the following examinations to determine whether you can participate in the study. The doctor will ask and record your medical history and perform a physical examination on you. You are diagnosed as a NAFLD patient by asking your medical history. You need to do B-ultrasound and blood tests to determine the degree of NAFLD. If you are a qualified enrollee, you can voluntarily participate in the study and sign an

informed consent form; if you are unwilling to participate in the study , We will treat according to your wishes.

(2) If you voluntarily participate in the study, the following steps will be taken

If you are fit and agree to participate in this study, you will be randomly assigned to the following two treatment groups:

1) LBP intervention group, 2) placebo group. LBP is a new type of prebiotic substance, extracted from wolfberry, with various functions such as immune regulation, anti-oxidation, and anti-tumor. [Specifications] 0.75g/tablet, [Manufacturer] Ningxia Wofu Bairui Biological Food Engineering Co., Ltd.

Before the intervention: All subjects need to sign an informed consent before the intervention. Before the intervention, the research subjects shall be intensively trained, the general demographic information of the participants shall be collected, and the feces and venous blood of the participants shall be collected, and the B-ultrasound diagnosis shall be performed and used Inbody body composition analyzer analyzes the patient's body composition, uses the International Physical Activity Questionnaire (IPAQ) to assess physical activity for a week, estimates nutritional intake through diet review, wears a watch for a week, and records daily physical activity, including exercise meter Step, heart rate, blood pressure, sleep, calorie detection, etc.

During the intervention: Participants in the intervention group were given wolfberry polysaccharide capsules, and participants in the control group received capsules containing polydextrose/maltodextrin with the same appearance as a placebo. One capsule a day, once a day, continue to be taken before meals during the intervention period. During the intervention period, the researchers followed up the two groups of patients once a month without changing their diet and daily physical activities.

Follow-up: At each follow-up, the participants will be given the capsules they need before the next follow-up. Use the capsule intake questionnaire and check the number of remaining capsules to assess the patient’s compliance, adverse events during the taking period, and whether to use other unconventional drugs. Will be recorded; the International Sports Activity Questionnaire (IPAQ) will be collected during follow-up, patients fill out once a week, and 3 food record forms will be collected.

After intervention: Concentrate within 48 hours after the trial intervention, collect 5ml of feces, urine, and peripheral venous blood of all participants, and conduct body composition analysis and B-ultrasound diagnosis.

If you cannot cooperate with treatment and follow-up during the study period, please inform us in time.

5. Possible adverse reactions, risks and treatment methods

Possible adverse reactions mainly include nausea, vomiting, loss of appetite, etc. When the above adverse reactions occur, if the severity is mild, no special treatment is required. If the symptoms are severe, they should be stopped immediately and given symptomatic treatment.

6. About the cost

We do not charge any additional fees for participating in this research. When a patient has an adverse reaction, the investigator will bear the cost of dealing with the adverse reaction.

7. What are the possible benefits of participating in the trial?

We hope to improve the treatment efficiency of NAFLD patients, improve the liver damage of NAFLD patients, slow down the progress of NAFLD, and provide guidance for the treatment of NAFLD in the future.

8. Will personal information be kept confidential?

Your medical records (research medical records, laboratory test reports, etc.) will be kept in the office of the School of Public Health and Management of Ningxia Medical University. Any information and data about you obtained during the trial will be kept strictly confidential, and the investigator, sponsor, supervisor authorized by the sponsor, the ethics committee of the center, and officials of the State Food and Drug Administration will be able to access Your personal information, but they will guarantee that they will not disclose your information to other parties. Although the research results may be published, they will not disclose your personal information in these publications.

9. Can voluntarily participate in research and opt out of research halfway

Whether or not to participate in this research is entirely out of your volition. If you decide to participate, you will be required to sign an informed consent form and a copy of this informed consent form will be obtained. If you participate in this study, you can still ask to withdraw at any time. If you withdraw, it will not affect your standard treatment.

Signed page of informed consent

Name of the clinical trial: Effect of Lycium barbarum polysaccharide supplementation in non-alcoholic fatty liver disease patients

project Undertaking unit: Ningxia Medical University

Subject statement

- I have read this informed consent form and have obtained relevant information about this trial. I have had the opportunity to ask the investigator for related questions about the clinical trial and have received answers.
- I know that I can withdraw from this trial at any time without suffering loss of profit or other adverse consequences.
- I am willing to cooperate with researchers in related examinations or treatments.
- I understand that my personal identity and privacy for participating in this research will be kept strictly confidential.
- I have obtained a copy of this informed consent form.

Subject's signature:_________________
Date:_____________________________
Tel: ______________________________

Signature of the legal representative:_________________________
(pleasespecify the direct relationship between the legal representative and the subject)
Date:____________________
Tel: _____________________

Statement from the investigator who performed the informed consent:

I or my research team have fully explained and explained to the subject the purpose of this clinical trial, the operation process, and the possible risks and potential benefits of the subject's participation in the trial, and satisfactorily answered the subject's relevant questions.

Investigator's signature:________________
date:_________________________________
Tel:_________________________________
