# Supplementary material for: Effect of Lycium barbarum polysaccharide supplementation in non-alcoholic fatty liver disease patients: study protocol for a randomized controlled trial
Source: Trials. 2021 Aug 26;22:566. doi: 10.1186/s13063-021-05529-6 (PMC8439032; doi:10.1186/s13063-021-05529-6)
Supplement: Supplementary file 3 — Additional file 3. Project Approval Notice of National Natural Science Foundation of China and Related Matters. (Project approval number: 81660537). [file 13063_2021_5529_MOESM3_ESM.pdf]

Project Approval Notice of National Natural Science Foundation of China and Related Matters

Yang Jianjun Mr/Ms:

According to the regulations of the National Natural Science Foundation of China and expert review opinions, the National Natural Science Foundation of China (hereinafter referred to as the Natural Science Foundation of China) decided to fund your application. Project approval number: 81660537, project name: Based on PGC-1α/Irisin/UCP1 pathways to explore lycium barbarum polysaccharide on regulatory mechanism of liver cell mitochondrial dysfunction during nonalcoholic fatty liver disease. Direct funding: 40 million. Starting and ending dates of the project: January 2017 to December 2020, the review opinions and revision opinions of the relevant projects were attached.

Please log in to the Science Foundation Network Information System (https://isisn.nsfc.gov.cn) as soon as possible to obtain the “National Natural Science Foundation of China Funding Project Plan" (hereinafter referred to as the plan) and fill in as required. For projects with revised opinions, please adjust the relevant content of the plan in time according to the revised opinions; if you have any objections to the revised opinions, you must submit it to the relevant science department before the deadline for submission of the electronic version of the plan. Note: please fill in the fund budget form of the plan in strict accordance with the "National Natural Science Foundation Funding Project Fund Management Measures". Among them, the amount listed in the subject of labor service fee and expert consultation fee shall not be increased compared with the application form.

The electronic version of the plan is uploaded through the Science Foundation Network Information System (https://isisn.nsfc.gov.cn), and then submitted to the Natural Science Foundation of China for review by the supporting unit. Those who fail the review should return to the revision before submitting; for those who pass the review, print the paper version of plan (in duplicate, double-sided printing). After being reviewed by the supporting unit and stamped with the official seal of the unit, submit the materials to the Natural Science Foundation of China Project Material Receiving Working Group. The content of the electronic version and the paper version of the plan shall be consistent.

The deadline for submitting the proposal is as follows:

1. At 16:00, September 11, 2016: The deadline for submitting the electronic version of the plan (considered as the official submission time of the plan);
2. At 16:00, September 18, 2020: Deadline for submitting the electronic revised version of

the plan;

3. At 16:00, September 26, 2020: Deadline for submitting the paper version of the plan.

Please submit the electronic version of the plan in time according to the above regulations, and submit the paper version of the plan. Those who fail to provide the reason and fail to submit the paper signed will be deemed to have automatically given up accepting funding.

Attachment: Project Review Opinion and Modification Opinion Form

National Natural Science Foundation of China

Department of Medical Sciences

August 17, 2016
