# Supplementary material for: Effect of Lycium barbarum polysaccharide supplementation in non-alcoholic fatty liver disease patients: study protocol for a randomized controlled trial
Source: Trials. 2021 Aug 26;22:566. doi: 10.1186/s13063-021-05529-6 (PMC8439032; doi:10.1186/s13063-021-05529-6)
Supplement: Supplementary file 4 — Additional file 4. Project Approval Notice of National Natural Science Foundation of China. (Project approval number: 82060597). [file 13063_2021_5529_MOESM4_ESM.pdf]

Project Approval Notice of National Natural Science Foundation of China

Yang Jianjun Mr/Ms:

According to the regulations of the National Natural Science Foundation of China and expert review opinions, the National Natural Science Foundation of China (hereinafter referred to as the Natural Science Foundation of China) decided to fund your application. Project approval number: 82060597, project name: Based on RhoA / ROCK signaling pathway to study the effect and mechanism of Lycium Barbarum Polysaccharide combined aerobic exercise on the intestinal barrier function of NAFLD. Direct funding: 35 million. Starting and ending dates of the project: January 2021 to December 2024, the review opinions and revision opinions of the relevant projects were attached.

Please log in to the Science Foundation Network Information System (https://isisn.nsfc.gov.cn) as soon as possible to obtain the “National Natural Science Foundation of China Funding Project Plan" (hereinafter referred to as the plan) and fill in as required. For projects with revised opinions, please adjust the relevant content of the plan in time according to the revised opinions; if you have any objections to the revised opinions, you must submit it to the relevant science department before the deadline for submission of the electronic version of the plan.

The electronic version of the plan is uploaded through the Science Foundation Network Information System (https://isisn.nsfc.gov.cn), and then submitted to the Natural Science Foundation of China for review by the supporting unit. Those who fail the review should return to the revision before submitting; for those who pass the review, print the paper plan (in duplicate, double-sided printing), rely on the unit to review and affix the official seal of the unit, and the paper signature and seal page of the application and set it at the back of one of the plans. Submit the above-mentioned materials to the Natural Science Foundation of China Project Materials Receiving Working Group. The content of the electronic version and the paper version of the plan shall be consistent. The Natural Science Foundation of China will review the paper signature and seal pages of the application form. If there are problems, the supporting unit is allowed to make one revision or supplement. Submit the paper signed and sealed page of the application form to the Natural Science Foundation of China. The deadline for submitting the proposal is as follows:

1. At 16:00, October 23, 2020: The deadline for submitting the electronic version of the plan (considered as the official submission time of the plan);

2. At 16:00, October 30, 2020: Deadline for submitting the electronic revised version of the plan;

3. At 16:00, November 06, 2020: Deadline for submitting the paper version of the plan (one of which contains the paper signature and seal page of the application).

4. At 16:00, November 27, 2020: Deadline for submitting the revised application form with paper signature and seal pages.

Please submit the electronic version of the plan in time according to the above regulations, and submit the paper version of the plan and application with paper signature and seal pages. Those who fail to provide the reason and fail to submit the paper signed and sealed pages of the plan or application form will be deemed to have automatically given up accepting funding. Those who fail to modify as required or submit the paper signature and seal page of the application form after the deadline will be subject to the suspension of payment of funds as appropriate.

Attachment: Project Review Opinion and Modification Opinion Form

National Natural Science Foundation of China

September 27, 2020
